# Diagnostic Accuracy of *H. pylori* Status by Conventional Endoscopy: Time-Trend Change After Eradication and Impact of Endoscopic Image Quality

**DOI:** 10.3389/fmed.2021.830730

**Published:** 2022-01-28

**Authors:** Duc Trong Quach, Rika Aoki, Akiko Iga, Quang Dinh Le, Toru Kawamura, Ken Yamashita, Shinji Tanaka, Masaharu Yoshihara, Toru Hiyama

**Affiliations:** ^1^Department of Internal Medicine, University of Medicine and Pharmacy at Hochiminh City, Ho Chi Minh, Vietnam; ^2^Department of Gastroenterology, Nhan Dan Gia Dinh Hospital, Ho Chi Minh, Vietnam; ^3^Department of Internal Medicine, Tokushima Health Screening Center, Tokushima, Japan; ^4^Department of Internal Medicine, Matsuyama-Joto Hospital, Matsuyama, Japan; ^5^Department of Internal Medicine, Kawamura Internal Medicine Clinic, Hiroshima, Japan; ^6^Department of Endoscopy, Hiroshima University Hospital, Hiroshima, Japan; ^7^Health Service Center, Hiroshima University, Higashihiroshima, Japan

**Keywords:** *Helicobacter pylori*, gastric atrophy, interobserver agreement, endoscopic diagnosis, Kyoto classification, Kimura-Takemoto classification

## Abstract

**Aim:**

To assess the time trend of diagnostic accuracy of pre- and post-eradication *H. pylori* status and interobserver agreement of gastric atrophy grading.

**Methods:**

A series 100 of conventional endoscopic image sets taken from subjects undergoing gastric cancer screening at a polyclinic were evaluated by 5 experienced assessors. Each assessor independently examined endoscopic findings according to the Kyoto classification and then determined the *H. pylori* status (never, current, or past infected). Gastric atrophy was assessed according to the Kimura-Takemoto classification and classified into 3 grades (none/mild, moderate, or severe). The image series that ≥3 assessors considered to have good quality were arbitrarily defined as high-quality image (HQI) series, and the rest were defined as low-quality image (LQI) series.

**Results:**

The overall diagnostic accuracy of *H. pylori* status was 83.0%. It was lowest in subjects with current infection (54%), gradually increased at 1 year (79%, *P* < 0.001) and 3 years (94.0%, *P* = 0.002), but then did not significantly change at 5 years (91.0%, *P* = 0.420) after eradication. The rate of LQI series was 28%. The overall diagnostic accuracy of *H. pylori* status dropped from 88.9% to 67.9% (*P* < 0.001), and the mean kappa value on gastric atrophy grading dropped from 0.730 to 0.580 (*P* = 0.002) in the HQI and LQI series, respectively.

**Conclusions:**

Diagnostic accuracy of *H. pylori* status increased over time after eradication. LQI series badly affected the diagnostic accuracy of *H. pylori* status and the level of agreement when grading gastric atrophy.

## Introduction

*Helicobacter pylori* (*H. pylori*) infection is the most consistent risk factor for gastric cancer (1). The cancer rarely develops in subjects never infected with *H. pylori*, and in infected patients, the risk level correlates with the severity and extent of gastric atrophy (2, 3).

*H. pylori* eradication is most beneficial for gastric cancer prevention before the development of severe precancerous lesions (1, 4). In a recent systematic review, the incidence of gastric cancer in patients with gastric atrophy ranged from 0.5 to 15.2 per 1000 person years, whereas there was more variation in gastric cancer incidence in patients with intestinal metaplasia (0.4 to 17.1 per 1000 person years) (5). Several cohort studies showed that gastric cancer still occurred after eradication in patients with severe and extensive atrophy, and these patients still need to receive endoscopic surveillance (6). The diagnosis of *H. pylori* infection status (*i.e*., current, past, or never infected) and grading of gastric atrophy are, therefore, important issues in the screening and surveillance for gastric cancer.

The endoscopic diagnosis of *H. pylori* status using the Kyoto classification of gastritis has been reported to have high accuracy (7, 8). Severe endoscopic gastric atrophy at baseline is an important risk factor for gastric cancer that has been well documented in the East and more recently in the West (9, 10). As endoscopic screening programs are recommended in regions with a high prevalence of gastric cancer, these two endoscopic judgements are important because they provide a real-time assessment of gastric cancer risk and possibly reduce the need for tests of *H. pylori* infection and mapping biopsy for histologic examination. These additional requirements could be more invasive and time-consuming and even lead to overload of the screening system, especially in regions with limited medical resource.

Currently, there is limited evidence regarding a time trend of the accuracy of endoscopic diagnosis of *H. pylori* status before and after eradication, and there are few studies on interobserver agreement of the endoscopic grading of gastric atrophy. Furthermore, as the endoscopic images used for these two endoscopic judgments in most of the previous studies were selected from teaching hospitals, the performance of these judgments using endoscopic images from real-life practice at general hospitals is unknown. The quality of endoscopic images could be an important factor that interferes with the translation of medical evidence into daily practice.

This study was conducted to assess both the time trend of the accuracy of diagnosis of *H. pylori* status by white light endoscopy (WLE) before and after *H. pylori* eradication and interobserver agreement of the endoscopic grading of gastric atrophy using an endoscopic image series from a polyclinic in Japan.

## Materials and Methods

### Materials

One hundred series of white-light gastroscopy images taken from subjects undergoing gastric cancer screening in Kawamura Internal Medicine Clinic, Hiroshima, Japan were retrospectively selected using the convenience sampling method (20 series from never-infected subjects and every 20 series from those with *H. pylori* infection obtained before and at 1, 3, and 5 years after successful eradication) using GIF-Q260 or GIF-H290 endoscopes (Olympus Co. Ltd., Tokyo, Japan). Series from subjects with localized lesions such as gastric cancer, those with a previous history of gastric surgery, and those who received proton pump inhibitors within 4 weeks prior to the esophagogastroduodenoscopy were excluded. All these endoscopic images were retrieved from the computerized database of the clinic. The size per image was approximately 1 Mb. Each standard image series consisted of 34 images: 5 of the prepylorus and antrum, 4 of the angulus, 21 of the corpus (9 look-ups and 12 look-downs), and 4 of the cardia and fornix.

The *H. pylori* status in each of these subjects was assessed with at least two of the following examinations: histology, serum or urine *H. pylori* antibody, and ^13^C-urea breath test. The sensitivity, specificity, and accuracy of the used urine test (Rapirun®Stick, Otsuka Pharmaceutical Co., Ltd, Japan) were 84.7, 89.9, and 87.0%, respectively (11). The image series taken from subjects who had all negative tests, and no history of *H. pylori* eradication were regarded as never-infected series. Those taken from subjects who had at least one positive test and no history of eradication were regarded as currently *H. pylori*-infected series. And those taken from subjects who had at least one positive test and had received eradication therapy with confirmed successful eradication results were considered as past-infected series.

### Assessors

All 100 of the image series were evaluated by 5 assessors, who were blinded to the patients' clinical information. All the endoscopic assessors had more than 7 years of experience including 3 Japanese (TH, RA, and AI) and 2 Vietnamese (DQ and QL). The two Vietnamese assessors have received 4-month training courses in Japan under the Japanese Society of Gastroenterology (JSGE) Research Fellowship Program focusing on endoscopic management of upper gastrointestinal malignancy.

**Figure 1:**
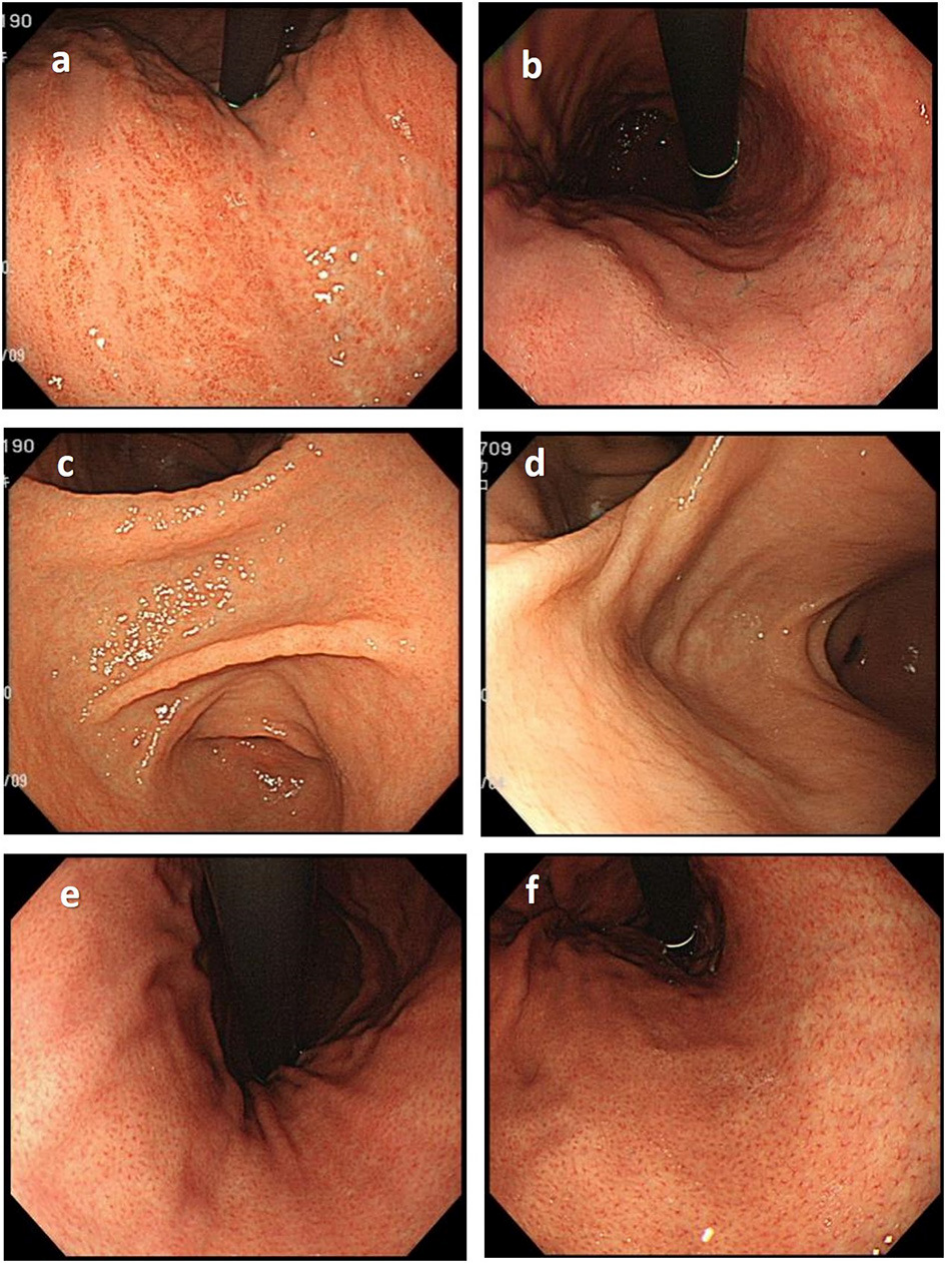
Important endoscopic findings and pitfalls in the correct diagnosis of *H. pylori* status. **(a)** Diffuse redness, typically seen in patients with current *H. pylori* infection, should be differentiated from patchy redness **(b)**, which can be seen in patients with past infection. **(c)** Mucosal edema is a typical finding commonly seen in current *H. pylori* infection that disappears in patients with past infection **(d)**. The regular arrangement of collecting venules **(e)**, which is a typical finding in the stomach of patients never infected with *H. pylori*, disappears in patients with *H. pylori* infection but could recur years after eradication **(f)**.

Before the endoscopic assessment, all assessors had attended a virtual seminar to discuss the endoscopic diagnosis of *H. pylori* status based on the Kyoto classification of gastritis and endoscopic assessment of gastric atrophy according to the Kimura-Takemoto classification (12, 13). During the seminar, the important findings as well as pitfalls in the correct diagnosis of *H. pylori* status were pointed out (Figure 1).

### Endoscopic Assessment of Image Series

#### *H. pylori* Status

The endoscopic features of gastritis according to the Kyoto classification were assessed in every image series, and the *H. pylori* status was decided based on the following criteria (7, 14, 15).

Non-infection: The presence of a regular arrangement of collecting venules observed in the normal gastric mucosa is the most important finding, and fundic gland polyps and red streaks are supporting findings.

Infection: Suggested findings include gastric atrophy, intestinal metaplasia, loss of a regular arrangement of collecting venules, xanthoma, hyperplastic polyp, and sticky mucus. Diffuse redness from the fundus to the stomach body and mucosal swelling are the most important findings indicating current infection. In addition, sticky mucus, enlarged folds, and nodularity can be seen. Map-like redness is a strong indication of past infection.

The assessors independently assessed the endoscopic findings of each image series and determined the *H. pylori* status based on their own general assessment.

#### Gastric Atrophy

Endoscopic gastric atrophy was assessed according to the Kimura-Takemoto classification. When the atrophic border touches the cardia ring even slightly, the atrophy grade was considered to be open-type (13). The severity of gastric atrophy was classified into 3 grades: none to mild (C-0 and C-1), moderate (C-2 to C-3), and severe (O-1 to O-3) (8, 15).

#### Endoscopic Image Quality

All assessors subjectively evaluated the overall quality of each image based on the difficulty of diagnosing the *H. pylori* status and assessing gastric atrophy. The causes of the low-quality image (LQI) series were classified into five categories: (1) insufficient air insufflation, (2) improper light (too dark or too bright), (3) blurred image, (4) retained mucus, bubbles, or food residue, and (5) poor color tone. The image series that 3 or more assessors considered to have good image quality were arbitrarily defined as high-quality image (HQI) series, and the rest were defined as LQI series.

### Statistical Analysis

The accuracies of the endoscopic diagnosis of *H. pylori* status were calculated for each assessor and all assessors in combination using the assessment results of all image series, the HQI and LQI series. They are presented as percentages with 95% confidence interval (CI) and compared using the χ2 test or Fisher's exact test as appropriate.

The interobserver agreement of gastric atrophy assessment was evaluated with kappa statistics. The results are presented as mean ± standard deviation (SD) and defined as follows: poor, ≤0.2; mild, 0.2 to 0.4; moderate, 0.4 to 0.6; good, 0.6 to 0.8; and excellent, 0.8 to 1 (15). The distribution of kappa values among assessors was checked for normal distribution using the Shapiro-Wilk test. The difference between mean kappa values using the HQI series and LQI series was evaluated using an independent sample *t*-test. JMP software (SAS Institute, Cary, NC, USA) was used for statistical analysis.

### Ethical Approval

The study protocol conforms to the ethical guidelines of the 1975 Declaration of Helsinki. This study was approved by the Ethical Committee of Hiroshima University (Approval Number: E-2003).

## Results

### Accuracy of Diagnosis of *H. pylori* Infection Status

**Figure 2:**
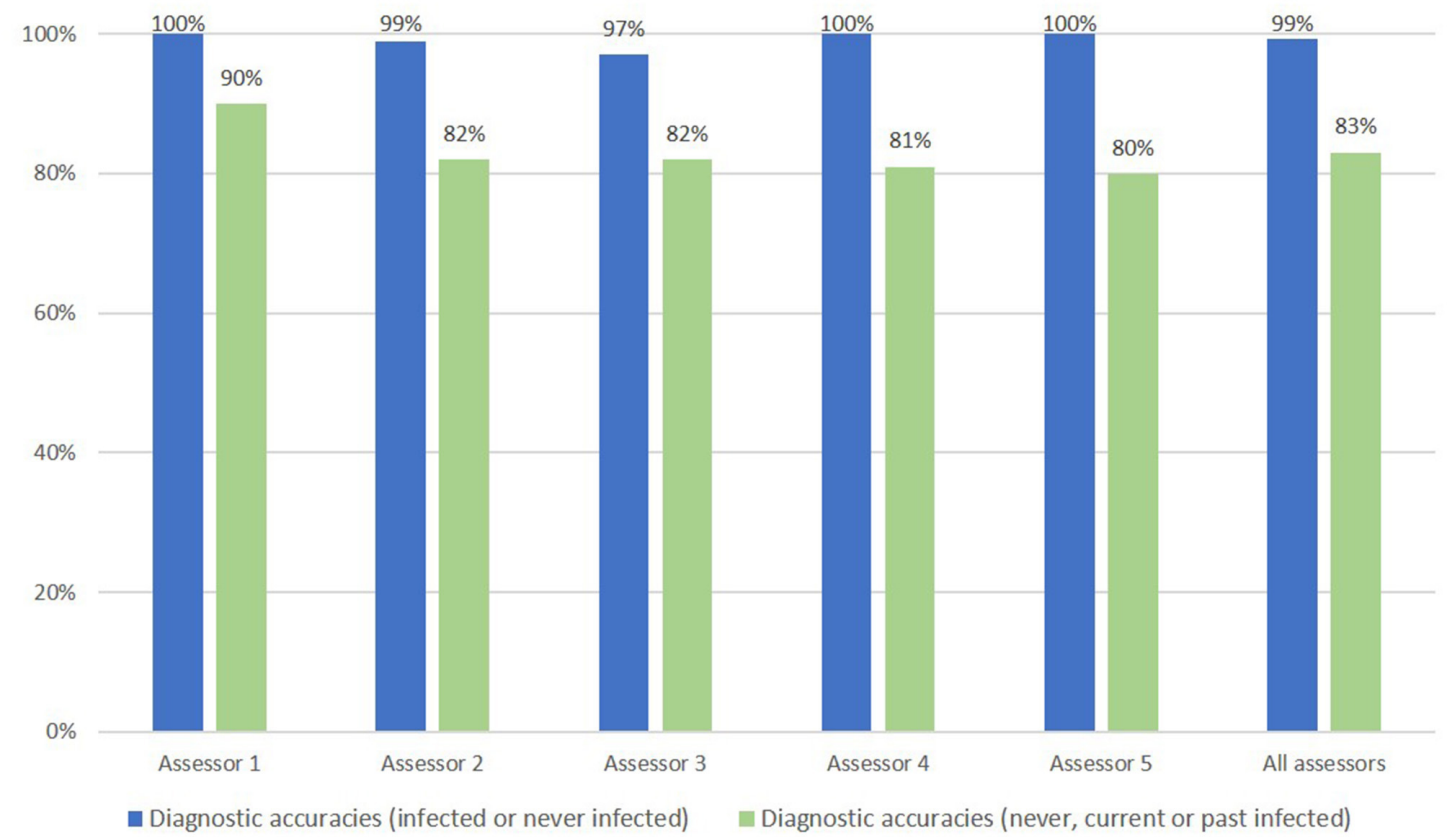
Endoscopic accuracy of *H. pylori* infection diagnosis (infected/never infected) and accuracy for *H. pylori* infection status (never, current, or past infected).

The accuracies of diagnosis of *H. pylori* infection (infected or never infected) by each assessor ranged from 97.0% (95% CI 91.5–99.4%) to 100% (95% CI 96.4–100%) (Figure 2). The accuracies of diagnosis of *H. pylori* status (never, current, or past infected) by each assessor were lower, ranging from 80.0% (95% CI 70.8–87.3%) to 90.0% (95% CI 82.4–95.1%). There were no significant differences in the accuracy of diagnosis of *H. pylori* status between the Japanese assessor group (assessors 1, 2, and 3) and the Vietnamese group (assessors 4 and 5). Combining the judgement results of all 5 assessors, the overall accuracies were 99.2% (95% CI 97.9–99.8%) (496/500) for the diagnosis of *H. pylori* infection (infected or never infected) and 83.0% (95% CI, 79.4–86.2%) (415/500) for the diagnosis of *H. pylori* status (never, current, or past infected).

**Figure 3:**
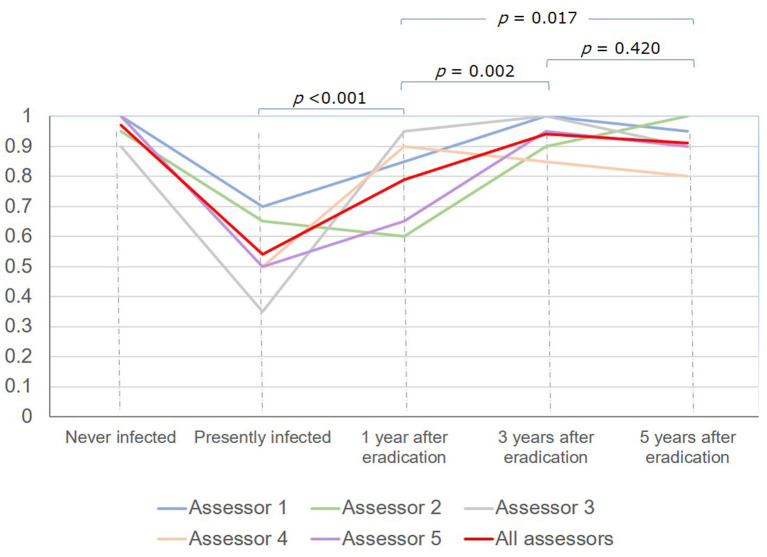
Time trend of diagnostic accuracy for *H. pylori* status based on white-light endoscopy.

The overall diagnostic accuracy for *H. pylori* status was lowest in subjects with current infection, gradually increased among subjects at 1 year and 3 years after eradication and did not significantly change thereafter: 54.0% (95% CI 43.7–64.0%), 79.0% (95% CI 69.7–86.5%), 94.0% (95% CI 87.4–97.8%), and 91.0% (95% CI 83.6–95.8%), respectively (Figure 3).

**Figure 4:**
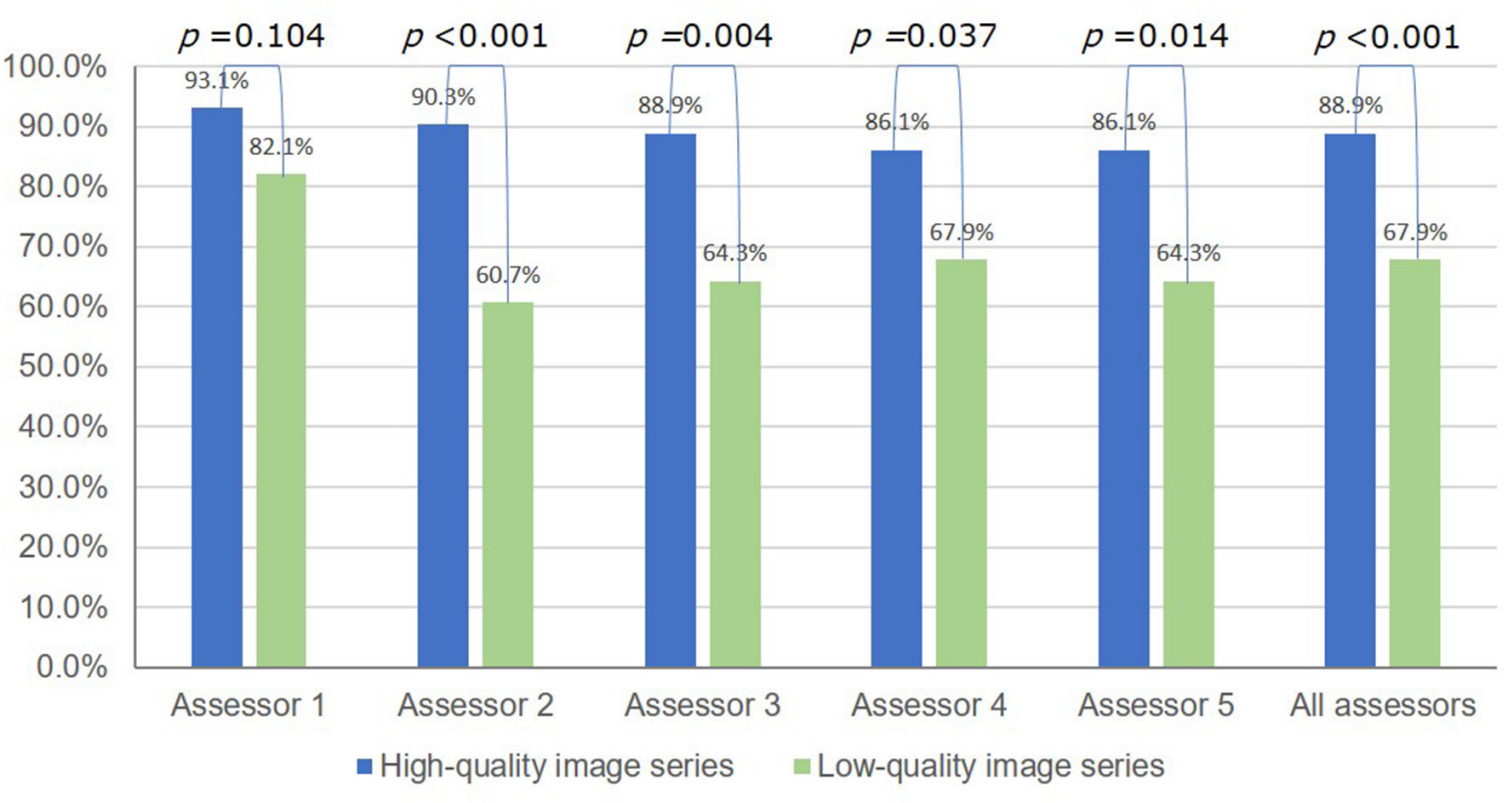
Accuracy of the endoscopic diagnosis of *H. pylori* infection status (never, current, or past infected) using high-quality vs. low-quality image series.

The overall diagnostic accuracies of *H. pylori* infection (infected or never infected) were excellent in both series with different image quality: 98.9% (95% CI 97.2–99.7%) (356/360) for the HQI series and 100% (95% CI 97.4–100%) (140/140) for the LQI series, respectively. However, the overall diagnostic accuracy of *H. pylori* status for the LQI series was significantly lower compared to that for the HQI series: 67.8% (95% CI 59.5–75.5%) (95/140) vs. 88.9% (95%CI, 85.2–91.9%) (320/360), respectively; *P* < 0.001 (Figure 4).

### Interobserver Agreement of Gastric Atrophy Grading

Table 1 shows the interobserver agreement of gastric atrophy grading using all image series, the HQI series, and the LQI series. The mean kappa value using all image series was 0.715 ± 0.084, and the value in the HQI series was significantly higher than that in LQI series (0.730 ± 0.090 vs. 0.580 ± 0.101, respectively; *P* = 0.002).

**Table 1:** Interobserver agreement of gastric atrophy grading using image series of different quality.

	**Assessor 1**	**Assessor 2**	**Assessor 3**	**Assessor 4**	**Assessor 5**
**Interobserver agreement using all image series**					
Assessor 1	-	0.800	0.722	0.766	0.746
Assessor 2	0.800	-	0.810	0.655	0.678
Assessor 3	0.722	0.810	-	0.545	0.631
Assessor 4	0.766	0.655	0.545	-	0.767
Assessor 5	0.746	0.678	0.631	0.767	-
**Interobserver agreement using high-quality image series**					
Assessor 1	-	0.829	0.769	0.768	0.762
Assessor 2	0.829	-	0.869	0.675	0.728
Assessor 3	0.769	0.869	-	0.563	0.641
Assessor 4	0.768	0.675	0.563	-	0.785
Assessor 5	0.762	0.728	0.641	0.785	-
**Interobserver agreement using low-quality image series**					
Assessor 1	-	0.646	0.511	0.734	0.678
Assessor 2	0.646	-	0.555	0.517	0.459
Assessor 3	0.511	0.555	-	0.446	0.571
Assessor 4	0.734	0.517	0.446	-	0.692
Assessor 5	0.678	0.459	0.571	0.692	-

### Endoscopic Image Quality

**Figure 5:**
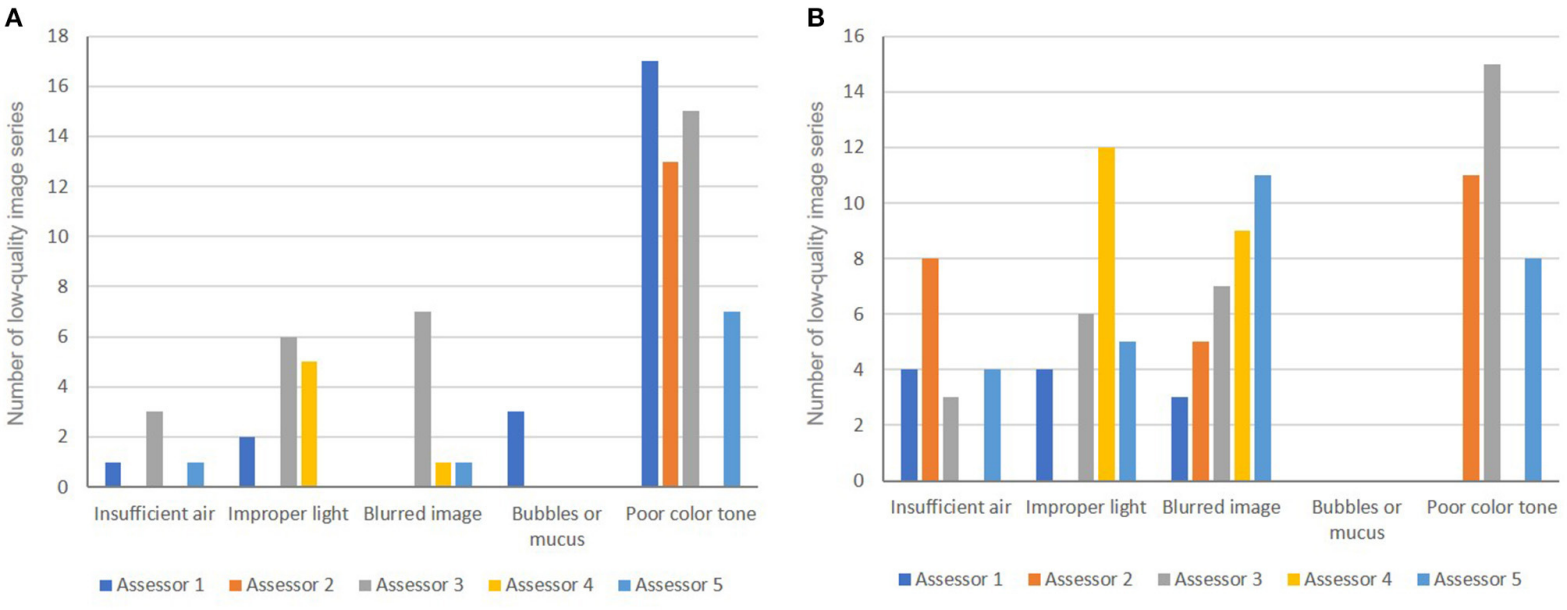
Causes of low-quality images used for the endoscopic diagnosis of *H. pylori* status **(A)** and for gastric atrophy grading **(B)**.

**Figure 6:**
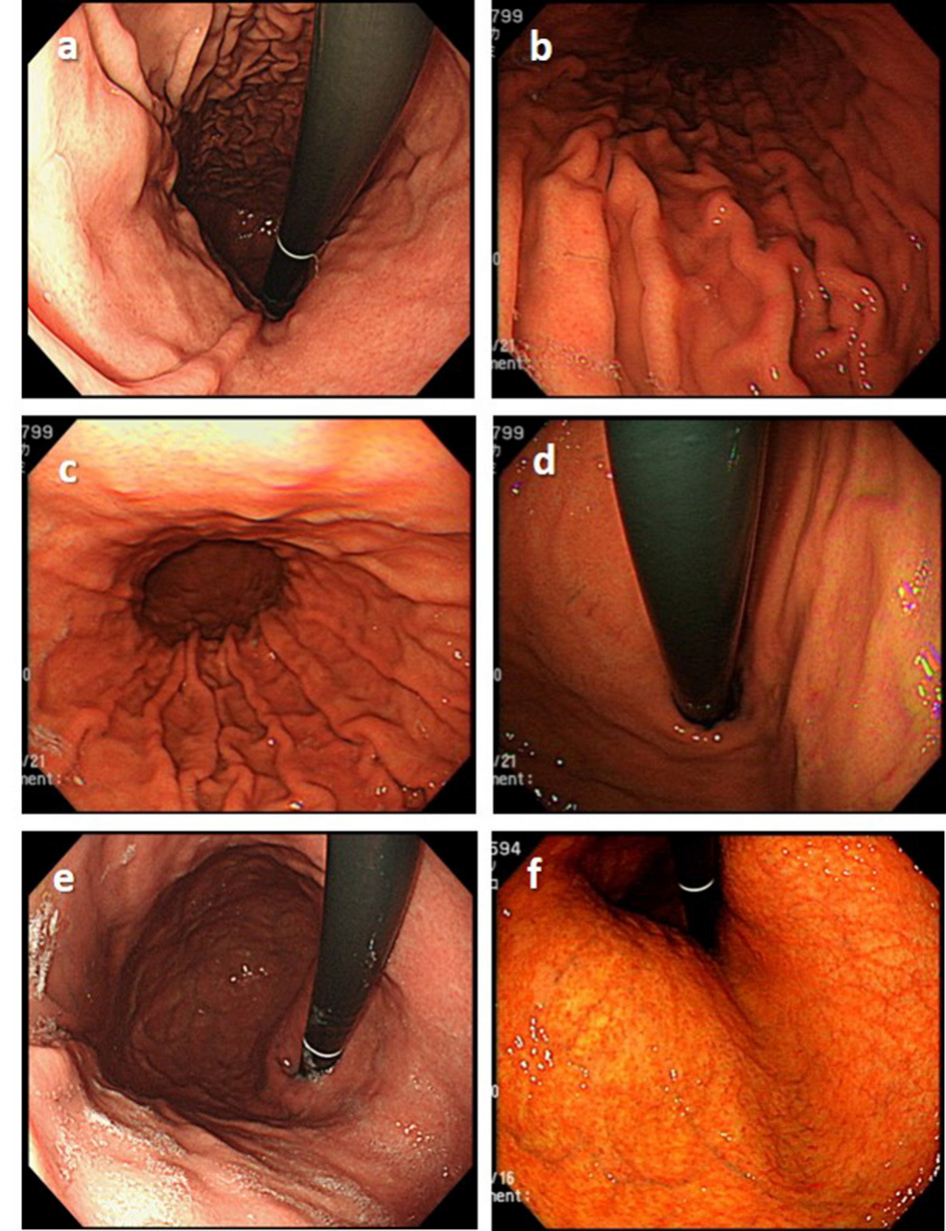
Examples of low-quality endoscopic images. **(a)** Insufficient air insufflation (image obtained in a patient at 4 years after eradication). **(b)** Improper light (too dark) (image obtained in a patient with current *H. pylori* infection). **(c)** Improper light (too bright) (image obtained in a patient with current *H. pylori* infection). **(d)** Blurred image (image obtained in a patient with current *H. pylori* infection). **(e)** Some mucus is still retained in the stomach (image obtained in a patient at 5 years after eradication). **(f)** Poor color tone (image obtained in a patient at 1 year after eradication).

The rate of LQI series was 28%. Poor color tone was the main factor that interfered with the endoscopic assessment of both *H. pylori* status and gastric atrophy (Figure 5). Blurred images, improper light, and air insufficiency were also contributing factors that made the assessment of gastric atrophy challenging (Figures 5, 6).

## Discussion

This study showed high accuracy of the endoscopic diagnosis of *H. pylori* status and good agreement on gastric atrophy grading using WLE in the hands of experienced endoscopists. It also showed for the first time, to our knowledge, that the diagnostic accuracy of *H. pylori* status increased over time after eradication, and that the LQI series significantly affected the accuracy of the endoscopic diagnosis of *H. pylori* status and the level of agreement on gastric atrophy grading.

Our results were similar to those reported by Yoshi et al., which showed that the assessor's general assessment based on WLE had very high accuracy for the endoscopic diagnosis of *H. pylori* status (7). In another study, Watanabe et al. reported a lower accuracy rate: 88.9% for never infected, 62.1% for currently infected, and 55.8% for *H. pylori*-eradicated subjects. This could be explained by the fact that the assessors in their study included both inexperienced and experienced assessors (16). Interestingly, our study showed similar accuracy of the endoscopic diagnosis of *H. pylori* status between the Japanese and Vietnamese endoscopists, which suggested that the Kyoto classification could be widely applicable in clinical practice outside of Japan with proper training.

Without knowledge of the history of *H. pylori* eradication, the most challenging issue in the endoscopic diagnosis of *H. pylori* status is to differentiate between current and past infection (7, 8). To our best knowledge, no studies have addressed the time trend of diagnostic accuracies of *H. pylori* status after eradication. Information regarding the time from eradication of endoscopic images used for endoscopic assessment in previous studies was not available. The present study showed that the diagnostic accuracy was lowest in subjects with current infection, increased gradually over time after eradication, reached a high rate of up to 94.0% at 3 years after eradication, and did not significantly change thereafter (Figure 3). The reasons may be as follows. Mucosal edema and diffuse redness, the important findings of current *H. pylori* infection (7, 14), may be overlooked under WLE. However, the color of gastric mucosa may become more whitish over time after eradication as diffuse redness disappears and leads to clearer map-like redness, a highly specific finding of past infection.

Image series with low quality would affect the accuracy of any endoscopic judgement. However, evidence regarding the impact of this issue on the diagnostic accuracy of *H. pylori* status and gastric atrophy assessment is lacking. By using image series from a polyclinic rather than teaching hospitals, this study showed that the LQI series significantly affected the translation of current evidence into daily practice. The overall diagnostic accuracy of *H. pylori* status significantly dropped from 88.9 to 67.9% (*P* < 0.001), and the mean kappa value for gastric atrophy grading dropped from good to moderate (0.730 vs. 0.580, *P* = 0.002) in HQI vs. LQI series, respectively. By comparing the endoscopic judgements between the two image series with different image quality, two important points for clinical practice were revealed. First, the excellent diagnostic accuracy of *H. pylori* status and good agreement on the grading of gastric atrophy in the HQI series strengthened the evidence supporting the application of the Kyoto and Kimura-Takemoto classifications in real-life practice. Second, that both endoscopic judgements were badly affected in the LQI series highlighted the importance of this issue. Adhered mucus, bubbles, and food residue are well documented to affect the quality of endoscopy, and pre-endoscopy preparation is strongly recommended in a recent international consensus (17). However, this was not the main cause of LQIs in this study, possibly because the image series had been strictly obtained with care regarding this issue. The assessors in this study raised other causes of concern, which included technical aspects such as poor color tone, improper light and operator-dependent concerns such as air insufflation and blurred images. The former would affect the real-time endoscopic assessment whereas the later would affect documentation for future purposes such as training and follow-up.

Image-enhanced endoscopy including magnifying endoscopy and digital chromoendoscopy such as narrow band imaging (NBI) and linked color imaging (LCI) offered advantages in diagnosing *H. pylori* (18). There are inconsistent findings regarding the diagnostic accuracy of *H. pylori* gastritis between NBI and WLI across studies (19, 20); but magnifying NBI and LCI have been shown to have higher diagnostic accuracy for the prediction of *H. pylori* status compared to WLE (21, 22). In addition, artificial Intelligence (AI) is also very promising diagnostic method as a recent meta-analysis reported that the accuracy of an AI algorithm for endoscopic diagnosis of *H. pylori* infection (never or past infection) reached 82% (23). Although its accuracy is still lower compared to that of experienced assessors, AI can help to shorten the learning curve for endoscopic diagnosis among beginners, and it may evolve further in the near future. In addition, other enhanced endoscopy techniques, especially magnifying narrow-band imaging and blue laser imaging-bright, can help to diagnose mucosal atrophy more accurately than WLE (24). These techniques promise a significant change in our practical approach in the future. However, WLE is currently the most widely available equipment in use for endoscopic screening. In regions with limited resources, the most applicable approach using WLE would be to check for the history of *H. pylori* eradication and to assess the endoscopic findings of *H. pylori* infection and gastric atrophy. As *H. pylori* self-eradication and reinfection are uncommon (25, 26), endoscopic findings suggesting *H. pylori* infection in subjects with no history of eradication likely indicate current infection. Additional tests for *H. pylori* infection diagnosis and a mapping biopsy would be needed only in a few difficult situations.

This study has some limitations. First, the criteria for *H. pylori* current infection included positive antibody tests, which were not reliable markers of current infection. Although spontaneous eradication of *H. pylori* is rare (25, 26) and the absence of a prior history of *H. pylori* eradication was required as a necessary criterion, the possibility of false-positive tests might not be accurately eliminated. Second, this is a single-center study with a limited number of assessors. Third, the materials used for the endoscopic assessment in this study were not video clips but still images. Finally, the time trend of diagnostic accuracy of *H. pylori* status in this study was only available for up to 5 years after eradication.

In conclusion, the present study showed that the diagnostic accuracy of *H. pylori* status by WLE increased over time after eradication. High accuracy in the endoscopic diagnosis of *H. pylori* status and good agreement on grading gastric atrophy could be achieved in the hands of experienced endoscopists. That LQI series, which might be not uncommon in daily practice, significantly affected both endoscopic judgments suggests that multi-center audits should be conducted to document the magnitude of this issue and to see whether standardized criteria for HQI series are required due to their potential impact on endoscopic screening for gastric cancer.

## Data Availability Statement

The raw data supporting the conclusions of this article will be made available by the authors, without undue reservation.

## Ethics Statement

The studies involving human participants were reviewed and approved by the Ethical Committee of Hiroshima University (E-2003). Written informed consent for participation was not required for this study in accordance with the national legislation and the institutional requirements.

## Author Contributions

DQ and TH initiated the study conception, designed the research study, assessed the endoscopic pictures, performed data analysis, and drafted the manuscript. RA, AI, and QL assessed the endoscopic pictures. TK prepared the endoscopic pictures. KY performed statistical analysis. ST and MY supervised the study. All authors contributed to the article and approved the submitted version.

## Conflict of Interest

The authors declare that the research was conducted in the absence of any commercial or financial relationships that could be construed as a potential conflict of interest.

## Publisher's Note

All claims expressed in this article are solely those of the authors and do not necessarily represent those of their affiliated organizations, or those of the publisher, the editors and the reviewers. Any product that may be evaluated in this article, or claim that may be made by its manufacturer, is not guaranteed or endorsed by the publisher.

## References

[1] Bang CS Lee JJ Baik GH. Artificial intelligence for the prediction of helicobacter pylori infection in endoscopic images: systematic review and meta-analysis of diagnostic test accuracy. J Med Internet Res. (2020) 22:e21983. 10.2196/21983 32936088 PMC7527948

[2] Chiu PWY Uedo N Singh R Gotoda T Ng EKW Yao K. An Asian consensus on standards of diagnostic upper endoscopy for neoplasia. Gut. (2019) 68:186–97. 10.1136/gutjnl-2018-317111 30420400

[3] Dohi O Majima A Naito Y Yoshida T Ishida T Azuma Y. Can image-enhanced endoscopy improve the diagnosis of Kyoto classification of gastritis in the clinical setting? Digestive Endoscopy. (2019) 32:191–203. 10.1111/den.13540 31550395

[4] Glover B Teare J Ashrafian H Patel N. The endoscopic predictors of Helicobacter pylori status: a meta-analysis of diagnostic performance. Therapeutic Adv Gastrointest Endosc. (2020) 13:2631774520950840. 10.1177/2631774520950840 33150333 PMC7586493

[5] Hiyama T Quach DT Le QD Ho LX Vu NHT Shimamoto F. Rate of Unintended Helicobacter pylori Eradication in the Vietnamese. Helicobacter. (2015) 20:156–7. 10.1111/hel.12210 25660825

[6] Haruma K Kato M Inoue K Murakami K Kamada T. Kyoto Classification of Gastritis. Tokyo: Nihon Medical Center. (2017).

[7] Kimura K Takemoto T. An Endoscopic Recognition of the Atrophic Border and its Significance in Chronic Gastritis. Endoscopy. (2008) 1:87–97. 10.1055/s-0028-109808626606158

[8] Mahachai V Vilaichone R-K Pittayanon R Rojborwonwitaya J Leelakusolvong S Maneerattanaporn M. Helicobacter pylorimanagement in ASEAN: the Bangkok consensus report. J Gastroenterol Hepatol. (2018) 33:37–56. 10.1111/jgh.13911 28762251

[9] Malfertheiner P Megraud F O'morain CA Atherton J Axon AT Bazzoli F. Management of helicobacter pylori infection–the maastricht IV/ florence consensus report. Gut. (2012) 61:646–664. 10.1136/gutjnl-2012-302084 22491499

[10] Maric L Castaneda D Singh H Bejarano P Jimenez Cantisano B Castro FJ. Kimura-takemoto classification: a tool to predict gastric intestinal metaplasia progression to advanced gastric neoplasia. Dig Dis Sci. (2021) 1–8. 10.1007/s10620-021-07212-x 34406583

[11] Miwata T Quach DT Hiyama T Aoki R Le HM Tran PLN. Interobserver and intraobserver agreement for gastric mucosa atrophy. BMC Gastroenterol. (2015) 15:1–6. 10.1186/s12876-015-0327-x 26239636 PMC4523036

[12] Ono S Dohi O Yagi N Sanomura Y Tanaka S Naito Y. Accuracies of Endoscopic Diagnosis of Helicobacter pylori -Gastritis: Multicenter Prospective Study Using White Light Imaging and Linked Color Imaging. Digestion. (2020) 101:624–30. 10.1159/000501634 31336366

[13] Pimentel-Nunes P Libânio D Lage J Abrantes D Coimbra M Esposito G. A multicenter prospective study of the real-time use of narrow-band imaging in the diagnosis of premalignant gastric conditions and lesions. Endoscopy. (2016) 48:723–30. 10.1055/s-0042-108435 27280384

[14] Quach DT. Value of a new stick-type rapid urine test for the diagnosis ofHelicobacter pyloriinfection in the Vietnamese population. World J Gastroenterol. (2014) 20:5087–91. 10.3748/wjg.v20.i17.5087 24803823 PMC4009545

[15] Quach DT Hiyama T. Assessment of endoscopic gastric atrophy according to the kimura-takemoto classification and its potential application in daily practice. Clin Endosc. (2019) 52:321–7. 10.5946/ce.2019.072 31327182 PMC6680010

[16] Rugge M Meggio A Pravadelli C Barbareschi M Fassan M Gentilini M. Gastritis staging in the endoscopic follow-up for the secondary prevention of gastric cancer: a 5-year prospective study of 1755 patients. Gut. (2019) 68:11–7. 10.1136/gutjnl-2017-314600 29306868

[17] Spence AD Cardwell CR Mcmenamin ÚC Hicks BM Johnston BT Murray LJ. Adenocarcinoma risk in gastric atrophy and intestinal metaplasia: a systematic review. BMC Gastroenterol. (2017) 17:1–9. 10.1186/s12876-017-0708-4 29228909 PMC5725642

[18] Sugano K Tack J Kuipers EJ Graham DY El-Omar EM Miura S. Kyoto global consensus report on Helicobacter pylori gastritis. Gut. (2015) 64:1353–67. 10.1136/gutjnl-2015-309252 26187502 PMC4552923

[19] Tongtawee T Kaewpitoon S Kaewpitoon N Dechsukhum C Loyd RA Matrakool L. Correlation between gastric mucosal morphologic patterns and histopathological severity ofhelicobacter pyloriassociated gastritis using conventional narrow band imaging gastroscopy. Biomed Res Int. (2015) 2015:1–7. 10.1155/2015/808505 26120585 PMC4450271

[20] Toyoshima O Nishizawa T Koike K. Endoscopic Kyoto classification of Helicobacter pylori infection and gastric cancer risk diagnosis. World J Gastroenterol. (2020) 26:466–77. 10.3748/wjg.v26.i5.466 32089624 PMC7015719

[21] Uemura N Okamoto S Yamamoto S Matsumura N Yamaguchi S Yamakido M. Helicobacter pylori infection and the development of gastric cancer. N Engl J Med. (2001) 345:784–9. 10.1056/NEJMoa001999 11556297

[22] Watanabe K Nagata N Shimbo T Nakashima R Furuhata E Sakurai T. Accuracy of endoscopic diagnosis of Helicobacter pyloriinfection according to level of endoscopic experience and the effect of training. BMC Gastroenterol. (2013) 13:1–7. 10.1186/1471-230X-13-128 23947684 PMC3765341

[23] Xiao S Fan Y Yin Z Zhou L. Endoscopic grading of gastric atrophy on risk assessment of gastric neoplasia: A systematic review and meta-analysis. J Gastroenterol Hepatol. (2021) 36:55–63. 10.1111/jgh.15177 32656803

[24] Yagi K Saka A Nozawa Y Nakamura A. Prediction of helicobacter pyloristatus by conventional endoscopy, narrow-band imaging magnifying endoscopy in stomach after endoscopic resection of gastric cancer. Helicobacter. (2014) 19:111–5. 10.1111/hel.12104 24372729

[25] Yang H Hu B. Diagnosis of helicobacter pylori infection and recent advances. Diagnostics. (2021) 11:1305. 10.3390/diagnostics11081305 34441240 PMC8391489

[26] Yoshii S Mabe K Watano K Ohno M Matsumoto M Ono S. Validity of endoscopic features for the diagnosis of Helicobacter pylori infection status based on the Kyoto classification of gastritis. Dig Endosc. (2020) 32:74–83. 10.1111/den.13486 31309632

